# Persistent organochlorine pesticides and cardiometabolic outcomes among middle-aged Latina women in a California agricultural community: The CHAMACOS Maternal Cognition Study

**DOI:** 10.1016/j.envint.2025.109302

**Published:** 2025-01-25

**Authors:** Marcella Warner, Stephen Rauch, Brenda Eskenazi, Lucia Calderon, Robert B. Gunier, Katherine Kogut, Nina Holland, Weihong Guo, Julianna Deardorff, Jacqueline M. Torres

**Affiliations:** a Center for Environmental Research and Community Health (CERCH), School of Public Health, University of California Berkeley, Berkeley, CA, USA; b Department of Epidemiology & Biostatistics, University of California San Francisco, San Francisco, CA, USA; c Division of Environmental Health Sciences, School of Public Health, University of California, Berkeley, Berkeley, CA, USA

**Keywords:** Obesity, Inflammation, Insulin resistance, Hypertension, Organochlorine pesticides, Dichlorodiphenyltrichloroethane

## Abstract

**Objective:**

Environmental exposure to endocrine disrupting compounds is hypothesized to increase risk of cardiovascular disease through effects on obesity, hypertension, dyslipidemia, and insulin resistance. We examined the relationship between serum concentrations of persistent organochlorine pesticides and biologic markers of inflammation and cardiometabolic disease, measured over a decade later, in a cohort of middle-aged and primarily immigrant Latina women living in an underserved agricultural community in California.

**Material and methods:**

We used data from the Center for the Health Assessment of Mothers and Children of Salinas-Maternal Cognition Study (CHAMACOS-MCS). We included 468 women who had concentrations of organochlorine pesticides measured in serum collected in 2009–2011 and complete follow-up data in 2022–2024 (blood draw, anthropometry, personal interview). We used Bayesian hierarchical regression models (BHM) to examine the independent effects of five highly correlated pesticides with continuous and binary measures of cardiometabolic disease and inflammation.

**Results:**

Participants averaged 49.0 (±5.5) years at follow-up. In BHM models, a 10-fold increase in *p,p*′-dichlorodiphenyltrichloroethane (DDT) and β-hexacyclohexane (β-HCH) was positively associated with BMI (DDT: adj-β = 1.26, 95 % Credible Interval (CrI): 0.33, 2.20; β-HCH: adj-β = 1.56, 95 %CrI: 0.45, 2.67) and waist circumference (DDT: adj-β = 2.75, 95 %CrI: 0.65, 4.85; β-HCH: adj-β = 3.74, 95 %CrI: 1.24, 6.23). Although credible intervals crossed the null, consistent positive associations were observed for DDT and β-HCH with blood pressure and for DDT with insulin resistance. Trans-nonachlor was positively associated with triglycerides (log-TRIG: adj-β = 0.08, 95 %CrI: 0.02, 0.13). β-HCH was positively associated with inflammatory markers (log-hsCRP: adj-β = 0.11, 95 %CrI: 0.03, 0.19; log-IL-6: adj-β = 0.08, 95 %CrI: 0.03, 0.14).

**Conclusion:**

With over a decade of follow-up, we extend evidence on previously reported associations of DDT and β-HCH with several measures of obesity. In addition, we provide new evidence suggesting associations with biomarkers of blood pressure, insulin resistance, dyslipidemia and inflammation, supporting the hypothesis that exposure may have long-term influences on cardiovascular disease risk.

## 1. Introduction

Cardiovascular diseases (CVDs), especially coronary heart disease and stroke, are the leading causes of death worldwide (Lozano et al., 2012), representing almost one-third of all global deaths (World Health Organization, 2021). Obesity is an important cardiovascular disease risk factor, frequently associated with a cluster of metabolic abnormalities including insulin resistance, hypertension, and dyslipidemia (Whitlock et al., 2009; Wormser et al., 2011). Environmental exposure to endocrine disrupting chemicals, including organochlorine pesticides, is hypothesized to increase risk of cardiovascular disease through effects on obesity, insulin resistance, hypertension, dyslipidemia, and inflammation (Casals-Casas and Desvergne, 2011; Heindel et al., 2017).

Organochlorine pesticides, including *p,p*′-dichlorodiphenyltrichloroethane (DDT), β-hexachlorocyclohexane (β-HCH), and hexachlorobenzene (HCB), are a group of legacy chemicals widely used in the United States from 1940 through the 1970s in agriculture and mosquito control (Stockholm Convention on Persistent Organic Pollutants (SCPOP), 2001; United Nations Environment Programme (UNEP), 2001). Under the Stockholm Convention, DDT continues to be used for public health purposes, such as for malaria control, in several countries, primarily in Sub-Saharan Africa (United Nations Environment Programme (UNEP), 2019). These pesticides are persistent in the environment, highly lipophilic, and bioaccumulate with relatively long half-lives ranging from six to 13 years in humans (Eskenazi et al., 2009; Jung et al., 1997; To-Figueras et al., 2000; Wolff et al., 2000). Although DDT has not been sprayed in the United States since 1972, most US residents still have detectable levels of the primary metabolite, *p,p*′-dichlorodiphenyldichloroethylene (DDE) (Centers for Disease Control and Prevention, 2009). Other global regions continued widespread use of DDT through the 1990s; US immigrants from these regions, including from Mexico and Central America, have been found to have higher levels of DDE (Muennig et al., 2011; Nguyen et al., 2020).

In experimental rodent studies, organochlorine pesticides are associated with a wide range of metabolic disruptive effects including visceral obesity, insulin resistance, and glucose intolerance (Ibrahim et al., 2011; Ruzzin et al., 2010; Tomatis et al., 1972). Results from epidemiologic evidence are less consistent and prospective studies are limited (Cano-Sancho et al., 2017; Hernández-Mariano et al., 2022; Peinado et al., 2020). Most epidemiologic studies consider only DDE exposure, and do not consider exposure to the complex mixture effects of organochlorine pesticides (Braun et al., 2016). Using data from the Center for the Health Assessment of Mothers and Children of Salinas (CHAMACOS) study (Eskenazi et al., 2003) of primarily immigrant Mexican-born women living in an underserved agricultural community in California, we previously reported significant positive associations between serum concentrations of DDT and β-HCH and several measures of body weight and composition (body mass index, obesity, waist circumference, percent body fat) assessed up to three years later (Warner et al., 2018). Here we examine the longitudinal relationship between serum concentrations of organochlorine pesticides and a wide range of biologic markers of inflammation and cardiometabolic disease measured over a decade later among the same cohort of women. We utilize Bayesian hierarchical regression models (BHM) to examine associations of individual organochlorine pesticides while accounting for correlated co-exposures (Hamra and Buckley, 2018).

## 2. Materials and methods

### 2.1. Study population

Data for this analysis comes from the Centers for the Health of Mothers and Children of Salinas-Maternal Cognition Study (CHAMACOS-MCS), a community-based cohort of mid-life women in California’s Salinas Valley who have been long-time participants in a study focused on environmental exposures and child health (Torres et al., 2024). Details of the CHAMACOS study have been presented elsewhere (Eskenazi et al., 2003). Briefly, between October 1999 and October 2000, pregnant women were recruited from prenatal clinics serving the farmworker population in the Salinas Valley, California. Eligible women were at least 18 years of age, less than 20 weeks gestation, English- or Spanish-speaking, qualified for government-sponsored health insurance, and planned to deliver at the county hospital. Of 525 women followed to delivery of a liveborn child, 348 women were still participating in the study as their index child turned 9 years (2009–2010). To refresh the cohort, in 2009–2011, 287 additional mother–child dyads were enrolled in the CHAMACOS study when their children were 9 years old. Eligibility criteria for both the original and refresher cohorts were similar to ensure that participants were from the same underlying population. Thus in total, 635 mother–child dyads participated in the CHAMACOS cohort study visit when their index child was 9Y (2009–2011). Of the 635 mothers, 572 (90 %) underwent a blood draw as part of the 2009–2011 study visit, in which serum concentrations of organochlorine pesticides were measured. Subsequent study visits were scheduled about every two years up until the index child was 18 years. Between May 2022 and July 2024, CHAMACOS mothers were recruited to participate in CHAMACOS-MCS, a new study focused on the mothers of the index children (Torres et al., 2024). Of the 635 eligible women, 577 women had updated contact information since 2014, of whom, 519 (90 %) women could be contacted and agreed to participate.

For the current analysis we included women who underwent a blood draw in 2009–2011 (in which organochlorine pesticides were measured) and participated in CHAMACOS-MCS in 2022–2024. Of the 519 women who agreed to participate, 473 had concentrations of organochlorine pesticides measured in serum collected in 2009–2011 and 468 women had complete anthropometric data collected in 2022–2024. For the laboratory outcomes, we excluded 14 women who did not undergo a blood draw in 2022–2024, leaving 454 women. All study activities were approved by the University of California, Berkeley, Office for the Protection of Human Subjects (Protocol ID 2021–02-14055) and written informed consent was obtained from all participants prior to participation.

### 2.2. Organochlorine pesticide exposure

Serum from fasting blood collected in 2009–2011 was stored at −80°C until shipment in 2013 (n = 378) and 2023 (n = 95) to the Centers for Disease Control and Prevention (Atlanta, GA) where specimens were analyzed for eight persistent organochlorine pesticides (*p,p*′-dichlorodiphenyltrichloroethane (DDT), *o,p*′-dichlorodiphenyltrichloroethane (*o,p*′-DDT), *p,p*′-dichlorodiphenyldichloroethylene (DDE), hexachlorobenzene (HCB), β-hexachlorocyclohexane (β-HCH), oxychlordane, *trans*-nonachlor, mirex) by gas chromatography isotope dilution high-resolution mass spectrometry (GC-ID-HRMS) (Jones et al., 2012). Method blanks (N = 3) and quality control (QC; N = 3) were included in each analytical batch of unknown (N = 24) samples. A measurement for a batch was considered valid if the following conditions were met: (i) the mean measured QC concentration fell within 3 standard deviations (STDs) from the established mean, (ii) the preceding two batches of QC measurements were within 2 STDs from the established mean, and (iii) a measurement within the preceding ten measurements had fallen above and below the established mean after a measurement had fallen above or below 2 STDs from the established mean. Total lipid content of each serum specimen was estimated using a “summation” method (Phillips et al., 1989), and analytical results were reported on a lipid-adjusted basis in units of nanograms per gram (ng/g) lipid. Quantifiable results less than the detection limits were reported when observed. For results below the LOD, a value was imputed based on a log-normal probability distribution via maximum likelihood estimation (Lubin et al., 2004). The analysis was restricted to pesticides with detection frequencies > 50 % and included DDT, DDE, HCB, β-HCH, and trans-nonachlor; oxychlordane (48.9 %), *o,p’-*DDT (5.5 %), and mirex (3.6 %) were excluded due to low frequency of detection.

### 2.3. Cardiometabolic outcome measures

Data collection for the CHAMACOS-MCS (2022–2024) included a fasting blood draw by venipuncture, anthropometric and blood pressure measurements, cognitive assessment and personal interview. Anthropometric measurements included height (cm), weight (kg), and waist circumference (cm). Barefoot standing height was measured to the nearest 0.1 cm using a wall-mounted stadiometer. Weight was measured to the nearest 0.1 kg using a bioimpedance scale (Tanita TBF-300A Body Composition Analyzer, Tanita Corporation of America, Inc. Arlington Heights, IL). Waist circumference was measured to the nearest 0.1 cm by placing a tape measure around the abdomen at the level of the navel and parallel to the floor; triplicate measures were made and averaged for analysis. We measured resting blood pressure at three 1-minute intervals using an automatic digital sphygmomanometer (Dianamap Carescape V100, Avante Health Solutions, Chicago IL) following American Heart Association recommendations; the last two measurements were averaged for analysis.

Cardiometabolic and inflammatory biomarkers including glucose, glycated hemoglobin (HbA1c), triglycerides, high-density lipoprotein (HDL) cholesterol, and high sensitivity C-reactive protein (hs-CRP) were measured in blood by LabCorp (Laboratory Corporation of America, Burlington, NC, USA). Inflammatory biomarker, interleukin-6 (IL-6), was measured in serum by enzyme-linked immunosorbent assay (ELISA, R&D Systems, Inc. Minneapolis, MN, USA) at the University of California Berkeley Public Health Biorepository.

We calculated body mass index (BMI, kg/m^2^) and women with a BMI ≥ 30 kg/m^2^ were classified as having obesity (World Health Organization 1998). Metabolic syndrome cases were diagnosed based on the presence of three or more of the following five criteria: 1) increased waist circumference ≥ 88 cm; 2) elevated triglycerides ≥ 150 mg/dL or report of current lipid-lowering medication; 3) low HDL-C < 50 mg/dL or report of current lipid-lowering medication; 4) increased blood pressure (systolic blood pressure ≥ 130 mm Hg, diastolic blood pressure ≥ 85 mm Hg or report of current antihypertensive medication); 5) elevated fasting glucose ≥ 100 mg/dL or report of current diabetes medication (Alberti et al., 2009). Diabetes cases were defined by self-report of doctor diagnosis on the interview (“Has a doctor ever told you that you had diabetes?) or current fasting glucose ≥ 126 mg/dL and/or current HbA1c ≥ 6.5 %. For inflammatory markers, participants with hs-CRP > 3 mg/dL were classified as high risk for cardiovascular disease (Pearson et al., 2003).

### 2.4. Covariates

At each study visit, women were interviewed in English or Spanish using a structured questionnaire. Information collected included individual and family sociodemographics and lifestyle characteristics, as well as pregnancy, reproductive, and medical histories. Covariates considered as potential confounders included age, country of origin, years of residence in the United States, primary language (Spanish or English), education level, employment status, marital status, household income, household food insecurity, smoking status, alcohol consumption, physical activity, parity, and menopause status. The final set of covariates was based on a directed acyclic graph (Supplementary Fig. 1) and included age (continuous variable, in years) at outcome assessment, and household income (categorical variable, at or below the federal poverty level versus above the federal poverty level) and years of residence in the U.S. (as a continuous variable) at the time of exposure assessment (i.e. at the time of the blood draw between 2009–2011).

### 2.5. Statistical analysis

Exposure variables all showed right-skewed distributions and were log_10_-transformed to reduce the influence of outliers and improve model fit. Distributions of triglycerides, hs-CRP, and IL-6 were also right-skewed; as a result, those outcomes were also log_10_-transformed in models using continuous outcomes. Models with continuous outcomes also contained the following exclusions based on other factors that may have affected their values: models of blood pressure outcomes (systolic, diastolic, mean arterial pressure, and pulse pressure) excluded 101 participants who self-reported taking medications to lower blood pressure at outcome assessment; models of HDL cholesterol and triglycerides excluded 60 participants who self-reported taking medications to lower cholesterol at outcome assessment; and models of glucose and HbA1c excluded 79 participants who self-reported taking diabetes medication at outcome assessment. These participants were included as cases in the related binary-outcome models.

To account for collinearity based on the high correlation between organochlorine exposures, we used semi-Bayesian Hierarchical regression models (BHMs) (Greenland, 1994; MacLehose et al., 2007; Thomas et al., 2007), fit by a 2-stage least-squares calculation (Greenland, 1997; Witte et al., 1998) to generate estimates. First, we ran models for each outcome including all exposures and covariates. We used linear regression to estimate associations with continuous outcomes and Poisson regressions with robust standard errors to estimate relative risks with binary outcomes (Zou, 2004). We then used coefficients from those models to create a linear weighted-least squares regression model based on the original coefficients and residual errors in order to produce estimates and credible intervals (CrI) (Witte et al., 2000). For continuous outcomes, we specified a prior variance equal to 1/3 of the interquartile range of each outcome. For binary outcomes, we specified a prior variance of 0.35 based on the assumption that the relative risks would be between 0.5 and 2. Estimates resulting from BHMs reflect the independent effect of a 10-fold increase in serum concentration of each organochlorine, adjusted for covariates and exposure to the remaining organochlorines. Due to the very high correlation between DDT and DDE, we did not attempt BHMs with both of these exposures in the same model. Rather, we ran two sets of models: one including DDT as well as HCB, β-HCH, and trans-nonachlor; and a second set including DDE as well as HCB, β-HCH, and trans-nonachlor. BHMs were created using R version 4.1.2; all other analyses were performed using Stata 17.0.

### 2.6. Sensitivity analysis

We repeated the final BHM models for laboratory measures (glucose, HbA1c, triglycerides, HDL, hs-CRP, IL-6) excluding the 66 (15 %) participants who underwent a non-fasting blood draw. We also repeated the final BHM models for continuous outcomes excluding outliers (>±3SD) for BMI (n = 8), waist circumference (n = 6), pulse pressure (n = 2), glucose and HbA1c (n = 5), HDL-C (n = 2), triglycerides (n = 4), hs-CRP (n = 2), and IL-6 (n = 7). Lastly, since many studies involving environmental exposures examine each exposure in separate models without accounting for other exposures, we ran separate single-exposure multivariable regression models for each exposure-outcome pair to allow for comparison with other studies.

## 3. Results

Select characteristics of the 468 participants are presented in Table 1. Participants were, on average, 36.5 (±5.4) years at the time of the exposure assessment (2009–2011) and 49.0 (±5.5) years at follow-up (2022–24) (Table 1). The majority of participants were born in Mexico (87.6 %) and arrived in the U.S. after age 18 (64.7 %; 23.6 (±4.2) years)). Compared to the baseline cohort, participants were less likely to be US-born (10.5 % versus 12.3 %). At the time of exposure assessment, participants had spent an average of 17.5 (±7.6) years of residence in the U.S., about three-quarters had not completed high school (77.0 %) and were living at or below the federal poverty level (74.2 %). At the time of outcome assessment, most women were married or living as married (71.8 %), multiparous (3.6 ± 1.3), and about one-third (38 %) were postmenopause.

Serum concentrations of persistent organochlorine pesticides, summarized in Table 2, were highly correlated (Supplementary Table 1). Geometric mean serum concentrations of DDT and DDE were 5.2 and 307.7 ng/g lipid, respectively, with DDE more than an order of magnitude higher and detected in over 99 % of samples. Trans-nonachlor, β-HCH and HCB were detected in 70–74 % of samples.

At the follow-up visit (2022–24), average BMI was 33.0 (±7.0) kg/m^2^, with 65.2 % of participants classified as obese and 87.0 % classified as having waist circumference ≥ 88 cm (Table 3). Among all participants, the prevalence of increased blood pressure (systolic blood pressure ≥ 130 mm Hg, diastolic blood pressure ≥ 85 mm Hg or report of current antihypertensive medication) was 38.5 % (n = 180). In total, 161 (34.0 %) participants met the diagnostic criteria for diabetes (self-report of a doctor diagnosis, current fasting glucose ≥ 126 mg/dL or HbA1c ≥ 6.5 %). A total of 220 (48.7 %) participants met the diagnostic criteria for metabolic syndrome; of those, 108 (49.0 %) met three criteria for diagnosis, 79 (35.9 %) met four criteria, and 33 (15.0 %) met all five criteria. The prevalence of elevated hs-CRP (i.e. > 3.0 mg/dL), was 46.8 %.In continuous BHM models (Table 4), a 10-fold increase in DDT and β-HCH was positively associated with BMI (DDT: adj-β = 1.26, 95 % Credible Interval (CrI): 0.33, 2.20; β- HCH: adj-β = 1.56, 95 % CrI: 0.45, 2.67) and waist circumference (DDT: adj-β = 2.75, 95 % CrI: 0.65, 4.85; β- HCH: adj-β = 3.74, 95 % CrI: 1.24, 6.23). Although credible intervals crossed the null, consistent positive associations were observed for DDT and β-HCH with blood pressure measures (systolic BP, diastolic BP, mean arterial pressure, pulse pressure) and for DDT with insulin resistance markers (glucose, HbA1c).

We observed mostly null associations of the other organochlorine pesticides with anthropometric measures and laboratory markers, with a couple of exceptions. A 10-fold increase in trans-nonachlor was positively associated with triglycerides (log-TRIG: adj-β = 0.08, 95 % CrI: 0.02, 0.13) and β-HCH was positively associated with inflammatory markers, hs-CRP (log-hsCRP: adj-β = 0.11, 95 % CrI: 0.03, 0.19) and IL-6 (log-IL-6: adj-β = 0.08, 95 % CrI: 0.03, 0.14).

The results of binary BHM models (Table 5) were consistent with continuous models. The RR for obesity was increased with a 10-fold increase in DDT (adj-RR = 1.11, 95 % CrI: 1.01, 1.23) and β- HCH (adj-RR = 1.16, 95 % CrI: 1.01, 1.33). The RR for elevated triglycerides was increased with a 10-fold increase in *trans*-nonachlor (adj-RR = 1.49, 95 % CrI: 1.11, 2.00). Although credible intervals crossed the null, the RR for increased waist circumference was associated with a 10-fold increase in DDT (adj-RR = 1.04, 95 % CrI: 0.98, 1.10) and β- HCH (adj-RR = 1.06, 95 % CrI: 0.99, 1.14), for metabolic syndrome was increased with a 10-fold increase in *trans*-nonachlor (adj-RR = 1.30, 95 % CrI: 0.99, 1.71), for diabetes was increased with a 10-fold increase in DDT (adj-RR = 1.16, 95 % CrI: 0.98, 1.39), and for elevated hs-CRP with a 10-fold increase in β-HCH (adj-RR = 1.18, 95 % CrI: 0.97, 1.44).

In sensitivity analyses, the results were similar (Supplementary Tables 2–4); in some cases, credible intervals shifted to include or exclude the null hypothesis, but associations remained in the same direction and credible intervals overlapped in every case. When we excluded participants with non-fasting blood (Supplementary Tables 2 and 3), the association of trans-nonachlor with triglycerides included the null (adj-β = 0.05, 95 % CrI: −0.01, 0.11). When we excluded outliers (>±3SD) (Supplementary Table 4), DDT was positively associated with HbA1c (adj-β = 0.10, 95 % CrI: 0.02, 0.17).

In continuous and binary BHM models, the estimates for HCB, β-HCH, and trans-nonachlor including the exposure mixture with DDE (Supplementary Tables 5 and 6) were almost identical to those reported including the exposure mixture with DDT (Tables 4 and 5). In single- exposure multivariable regression models, results were also substantively similar (Supplementary Tables 7 and 8). In addition, DDT was positively associated with HbA1c (DDT: adj-β = 0.17, 95 % Confidence Interval (CI): 0.02, 0.32), β-HCH was associated with a higher risk of waist circumference ≥ 88 cm (adj-RR = 1.07, 95 % CI: 1.01, 1.14) and trans-nonachlor was associated with a higher risk for metabolic syndrome (adj-RR = 1.30, 95 % CI: 1.01, 1.67).

## 4. Discussion

In this novel prospective cohort study of middle-aged and primarily immigrant Latina women residing in an underserved agricultural community in California, we examined the relationship between serum concentrations of persistent organochlorine pesticides and a wide range of biologic markers of inflammation and cardiometabolic disease measured over a decade later. We confirm our earlier report of associations with obesity measured about three years after exposure assessment (Warner et al., 2018), finding that these associations hold more than a decade later. We also extend these previous findings to consider a broader range of outcomes, including insulin resistance, hypertension, dyslipidemia, and inflammation. With over a decade of follow-up, we found higher serum levels of DDT and β-HCH were associated with higher BMI and risk of obesity, as well as larger waist circumference. We observed consistent positive associations, although values crossed the null, for both DDT and β-HCH with blood pressure, and for DDT with insulin resistance. Serum β-HCH levels were also positively associated with inflammatory markers, hs-CRP and IL-6. We observed mostly null associations for the other organochlorine pesticides with one exception; serum *trans*-nonachlor levels were associated with higher serum triglycerides.

To our knowledge this is the first study to evaluate associations of these highly correlated persistent organochlorine pesticides with a wide range of cardiometabolic risk factors and outcomes in a predominantly Latina population. The prior literature is generally spread across a wide range of studies that each focus on a subset of the outcomes included in our analysis, although some outcomes (e.g. dyslipidemia, inflammatory markers) have been particularly understudied. Moreover, there has been a great deal of variation across prior work in terms of study population (e.g. age, country-of-origin), which organochlorine pesticides are measured, the timing of exposure and outcome assessment, and other methodological details (e.g. single pesticide estimates versus considering the presence of other, highly correlated pesticides).

Our findings are consistent with some, but not all, previous prospective epidemiologic studies of organochlorine pesticides and BMI and waist circumference. In the Child Health and Development Study (CHDS), in utero DDT (but not DDE) exposure was positively associated with BMI and waist circumference in middle-aged daughters (La Merrill et al., 2020). Similarly, serum concentrations of DDT and DDE (but not β-HCH, HCB, or trans-nonachlor) were positively associated with BMI and waist circumference 20 years after exposure assessment in a population-based study of middle-aged adults (CARDIA) (Lee et al., 2011b). In contrast, among participants of the Prospective Investigation of the Vasculature in Uppsala Seniors (PIVUS) study serum levels of DDE, HCB, and trans-nonachlor were not associated with waist circumference five years later (Lee et al., 2012). However, neither DDT nor β-HCH were measured and given the high correlation of these pesticides, associations observed may be surrogate markers for other unmeasured chemicals.

Prior studies focused on other outcomes have been similarly mixed. Our results for the relationship of DDT and β-HCH and blood pressure are somewhat consistent with the limited previous prospective studies. In the CHDS, in utero DDT exposure was associated with self-report of hypertension among daughters in adulthood (La Merrill et al., 2013a). Adipose tissue concentrations of β-HCH, as well as DDE and HCB, were associated with increased risk of hypertension, albeit non-significantly, in a cohort of Southern Spain residents over a 10-year follow-up (Arrebola et al., 2015). On the other hand, in the Northern Sweden Health and Disease study, plasma concentrations of HCB and DDE were not associated with odds of hypertension among middle-age adults after 10-years of follow-up, but DDT and β-HCH were not measured (Donat-Vargas et al., 2018).

A similar mix of positive and null associations have been reported between DDT exposure and diabetes. Significant associations of serum DDE with diabetes were reported in a Great Lakes sport fish consumers cohort over a 10-year period (Turyk et al., 2009) and a nested case-control study within the Women’s Health In the Lund Area (WHILA) cohort (Rignell-Hydbom et al., 2009), whereas no association was found in the PIVUS study (Lee et al., 2011a) or nested case-control studies within the Nurse’s Health Study II (Zong et al., 2018) or the CARLA / KORA study in Germany (Wolf et al., 2019). While the majority of studies assessed DDE exposure, DDT exposure was measured in just three case-control studies. In a hospital-based case control study in China, significant positive associations were found for diabetes with all organochlorine pesticides measured including DDT, DDE, β-HCH, and *trans*-nonachlor (Han et al., 2020). In contrast, no associations were found for diabetes with serum DDT or DDE in nested case-control studies within CARDIA (Lee et al., 2010) or the Nurse’s Health Study (Wu et al., 2013) after 18 years of follow-up.

Cardiovascular disease risk factors including dyslipidemia and inflammation have been particularly understudied in relation to persistent organochlorine pesticides exposure. Only one other prospective study has evaluated associations with dyslipidemia, with findings generally aligned with ours; in the CARDIA study serum levels of trans-nonachlor were associated with higher triglycerides and lower HDL-C, 20 years after exposure assessment (Lee et al., 2011b). A small number of cross-sectional studies have evaluated associations with inflammatory markers, reporting findings consistent with our longitudinal associations. Serum concentrations of β-HCH (and trans-nonachlor but not DDE) were associated with increased CRP levels in NHANES (Kim et al., 2012). Serum β-HCH concentrations (but not DDT or DDE) were also positively correlated with IL-6 levels among women who had delivered a term birth in India (Mustafa et al., 2015). In a Swedish population-based study (PIVUS), no associations of inflammatory markers, IL-6 or CRP, were found with serum concentrations of DDE, HCB, or trans-nonachlor; unfortunately β-HCH was not measured.

Our results are biologically plausible. In animal studies, exposure to low doses of DDT has been associated with increased weight gain, abdominal obesity and insulin resistance (Ibrahim et al., 2011; Ruzzin et al., 2010; Tomatis et al., 1972). Various mechanisms of action are likely involved in chemical-induced adipogenesis and insulin resistance, and the mechanisms may differ among organochlorine pesticides (La Merrill et al., 2013b). Animal studies suggest exposure to DDT might decrease pancreatic secretory activity leading to impaired insulin secretion (Yau and Mennear, 1977), and disturb the regulation of thermogenesis, lipids, and glucose, which could lead to insulin resistance and metabolic alterations (La Merrill et al., 2014). *In vitro*, DDT has been shown to alter adipocyte differentiation (Moreno-Aliaga and Matsumura, 2002) and impair glucose metabolism and induce insulin resistance via disruption of lipid homeostasis (Ruzzin et al., 2010). Experimental evidence also supports the role of organochlorine pesticides in the development of hypertension, dyslipidemia, and inflammation; DDT exposure has been shown to cause an over-activation of the renin angiotensin system, which could produce a rise in blood pressure (La Merrill et al., 2016). Trans-nonachlor exposure was shown to produce an increase in intracellular neutral lipid accumulation associated with increased intracellular triglycerides (Howell et al., 2018). β-HCH exposure was shown to invoke a marked neuro-inflammatory response inducing up-regulation of pro-inflammatory cytokines, including IL-6, both *in vitro* and *in vivo* (Grieco et al., 2024; Shah et al., 2020).

The strengths of this study include its relatively large sample size, understudied Latina study population, and longitudinal design with a long follow-up period. We were able to consider a wide array of biomarkers of cardiometabolic disease and inflammation including multiple measures of anthropometry and blood pressure, as well as laboratory measures including glucose, HbA1c, triglycerides, HDL cholesterol, hs-CRP, and IL-6 in fasting blood. In addition, previous epidemiologic studies have only considered exposure to a single chemical at a time, which may not address the true effect of individual chemicals within a mixture. By using BHM, we were able to examine associations of individual organochlorine pesticides while accounting for correlated coexposures. Limitations of the study include exposure was measured in blood collected at one point in time, however, given the persistence and long half-life of these compounds, we would expect the rank order of exposure to be similar. No data were available regarding diet which may be relevant for both exposure and outcomes. Lastly, we did not measure exposure to other persistent organochlorine compounds such as polychlorinated biphenyls, dioxins or furans, which are ubiquitous at low levels in the environment and may be associated with cardiometabolic outcomes (Warner et al., 2013).

Although the serum concentrations of DDT, DDE, and β-HCH among CHAMACOS participants were higher than the general U.S. population levels (Centers for Disease Control and Prevention, 2009), our findings are relevant internationally. The majority of CHAMACOS-MCS participants are immigrants from Mexico, where they may have been directly exposed to DDT, which was restricted for malarial control use in 1991 and banned in 2000, as well as to HCB and β-HCH, which were used until the early 1990 s (Chanon et al., 2003; Li, 1999). However, DDT continues to be used in at least 13 countries around the world and given the long half-life of these organochlorine compounds, populations world- wide continue to have measurable levels in their blood and to be exposed from residues in the environment (Eskenazi et al., 2009; United Nations Environment Programme (UNEP), 2019).

In the United States, the prevalence of obesity and related cardiometabolic outcomes varies by race and ethnicity, with a higher prevalence among Latino populations (Flegal et al., 2016; Ford et al., 2010; Menke et al., 2015; Schneiderman et al., 2014). Given that the CHAMACOS population was more highly exposed than the general US population of Latinos (Centers for Disease Control and Prevention, 2009), it remains possible that exposure to these organochlorine compounds may be one factor in the even higher prevalence of obesity, diabetes, and metabolic syndrome observed in this population than in the general US Latino population (Flegal et al., 2016; Ford et al., 2010; Menke et al., 2015).

## 5. Conclusion

In summary, in this novel cohort study of middle-aged and primarily immigrant Latina women residing in an underserved agricultural community in California, we found significant associations of individual organochlorine pesticides, in particular DDT and β-HCH, with biomarkers of cardiometabolic disease and inflammation, which were independent of other organochlorine pesticide exposures. These results provide support for the hypothesis that long term exposure may increase risk of cardiovascular disease later in life.

## Supplementary Material

Supplementary data

Appendix A. Supplementary data

Supplementary data to this article can be found online at https://doi.org/10.1016/j.envint.2025.109302.

## Figures and Tables

**Table 1:** Select characteristics of participants, CHAMACOS Maternal Cognition Study, Salinas, California.

Characteristic	N (%)
**Total**	468 (100.0)
**Age at outcome assessment (years)**, mean ± SD	49.0 ± 5.5
**Race/ethnicity**	
Latina	452 (96.6)
Non-Latina	16 (3.4)
**Nativity**	
Mexico	410 (87.6)
US	49 (10.5)
Other	9 (1.9)
**Nativity/Age at first migration**	
Born in US	49 (10.5)
Immigrant, arrived in US as child (≤ 17 years)	116 (24.8)
Immigrant, arrived in US as adult (≥ 18 years)	303 (64.7)
**Time in US at exposure assessment (years)**, mean ± SD	17.5 ± 7.6
**Primary language**	
Spanish	415 (88.7)
English	53 (11.4)
**Educational attainment**	
≤ 6th grade	204 (43.6)
7–12th grade	156 (33.4)
≥ HS graduate	108 (23.1)
**Household Income at exposure assessment**	
At or below FPL	347 (74.2)
≥ FPL	121 (25.8)
**Marital status at outcome assessment**	
Married / living as married	336 (71.8)
Not married	132 (28.2)
**Parity at outcome assessment**	
1–2	85 (18.2)
3	163 (34.8)
4+	220 (47.0)
**Menopause status at outcome assessment**	
Premenopause	290 (62.0)
Postmenopause	178 (38.0)

Source: Center for the Health Assessment of Mothers and Children of Salinas (CHAMACOS) Maternal Cognition Study. Exposure assessment was completed in 2009–2011; outcome assessment was completed in 2022–2024. Abbreviations: FPL, Federal Poverty Level.

**Table 2:** Summary of persistent organochlorine pesticide concentrations (ng/g lipid) measured in serum collected in 2009–2011, CHAMACOS Maternal Cognition Study, Salinas CA, 2022–2024.

Exposure	N	% Detect	% Quant.	GM	Min	25 %	Med	75 %	Max
*p,p*′-DDT	468	66.4	100.0	5.2	0.4	2.0	3.5	8.1	5,276.5
*p,p*′-DDE	467	99.8	99.8	307.7	5.8	132.7	238.9	559.0	55,836.0
HCB	467	71.1	99.8	10.9	1.2	7.5	10.2	15.5	349.7
β-HCH	466	70.2	99.6	5.5	0.1	2.3	5.6	13.5	764.6
Trans-nonachlor	467	74.1	99.8	4.2	0.6	2.6	3.9	6.7	263.4

Abbreviations: *p,p*′-DDT, *p,p*′-dichlorodiphenyltrichloroethane; *p,p*′-DDE, *p,p*′-dichlorodiphenyldichloroethylene; HCB, hexachlorobenzene; β-HCH, β-hexachlorocyclohexane.

**Table 3:** Summary of Cardiometabolic and Inflammatory Markers, CHAMACOS Maternal Cognition Study, Salinas, CA 2022–2024.

	n (%) or mean ± SD
**Total** ^a^	468 (100.0)
**Anthropometry**	
Body mass index (kg/m^2^)	33.0 ± 7.0
Overweight (25-<30 kg/m^2^)	123 (26.3)
Obese (≥30 kg/m^2^)	305 (65.2)
Waist circumference (cm)	104.1 ± 15.6
Waist circumference ≥ 88 cm	407 (87.0)
**Blood pressure**	
Systolic blood pressure (mmHg)^b^	118.8 ± 13.5
Diastolic blood pressure (mmHg)^b^	65.3 ± 8.0
Mean arterial pressure (mmHg)^b^	86.6 ± 9.4
Pulse pressure (mmHg)^b^	53.5 ± 9.7
Increased blood pressure^c^	180 (38.5)
**Lipids**	
Serum triglycerides (mg/dL)^d h^	133.5 ± 1.60
Elevated triglycerides (≥150 mg/dL or drug tx)	202 (44.6)
Serum HDL cholesterol (mg/dL)^d,^	50.2 ± 11.3
Low HDL cholesterol (<50 mg/dL or drug tx)	265 (58.5)
**Insulin resistance**	
Serum glucose (mg/dL)^e^	95.8 ± 29.1
Elevated glucose (≥100 mg/dL or drug tx)	159 (35.2)
HbA1c (%)^e^	5.9 ± 0.9
Elevated HbA1c (≥ 6.5 %)	87 (19.3)
Diabetes^f^	161 (34.0)
Metabolic Syndrome^g^	220 (48.7)
**Inflammation**	
hs-CRP (mg/dL)^h^	2.67 ± 2.92
Elevated hs-CRP (>3.0 mg/dL)	212 (46.8)
IL-6 (pg/mL)^h^	2.15 ± 2.08

Abbreviations: HbA1c, glycated hemoglobin; HDL-C, high-density lipoprotein cholesterol; hs-CRP, high sensitivity C-reactive protein; IL-6, interleukin 6; tx, treatment.

a Total with measurements (n = 468); Total with blood (n = 454): TG (n = 452), HDL (n = 452), glucose (n = 452), HbA1c (n = 452), IL-6 (n = 454), hsCRP (n = 453).

b Exclude 101 (21.6 %) participants who report current medication for hypertension (n = 367).

c Increased blood pressure defined by SysBP ≥ 130 mmHg or DiaBP ≥ 85 mmHg or drug tx.

d Exclude 60 (13.2 %) participants who report current cholesterol lowering medication (n = 393).

e Exclude 79 (17.4 %) participants who report current medication for diabetes (n = 374).

f Diabetes cases defined by fasting glucose ≥ 126 mg/dL or HbA1c ≥ 6.5 % or self-report.

g Metabolic Syndrome cases diagnosed based on the presence of ≥ 3 factors: (1) waist circumference ≥ 88 cm; (2) serum triglycerides ≥ 150 mg/dL or drug treatment to lower cholesterol; (3) serum HDL-C < 50 mg/dL or drug treatment to lower cholesterol (4) systolic blood pressure ≥ 130 mm Hg or diastolic blood pressure ≥ 85 mm Hg or drug treatment for high blood pressure; (5) serum glucose ≥ 100 mg/dL or drug treatment for diabetes.

h Geometric mean (±SE).

**Table 4:** Adjusted^a^ associations between OC pesticide exposure (log_10_) and continuous cardiometabolic and Inflammatory outcomes using linear semi-Bayesian Hierarchical Models, CHAMACOS Maternal Cognition Study, Salinas, CA 2022–2024.

Outcome	n^c^	*p,p*′-DDT β (95 % CrI)	*p,p*′-DDE^b^ β (95 % CrI)	HCB β (95 % CrI)	β-HCH β (95 % CrI)	Trans-nonachlor β (95 % CrI)
Body Mass Index (kg/m^2^)	468	1.26 (0.33, 2.20)*	0.57 (−0.56, 1.70)	−0.24 (−2.03, 1.56)	1.56 (0.45, 2.67)*	−0.25 (−1.98, 1.48)
Waist Circumference (cm)	468	2.75 (0.65, 4.85)*	1.18 (−1.35, 3.71)	0.42 (−3.62, 4.46)	3.74 (1.24, 6.23)*	0.65 (−3.24, 4.54)
Systolic Blood Pressure (mmHg)	367	0.74 (−1.32, 2.79)	−0.39 (−2.85, 2.06)	−0.46 (−4.47, 3.56)	0.85 (−1.66, 3.36)	−0.65 (−4.50, 3.20)
Diastolic Blood Pressure (mmHg)	367	0.32 (−0.94, 1.57)	−0.36 (−1.87, 1.15)	−0.86 (−3.38, 1.67)	0.44 (−1.10, 1.98)	−0.07 (−2.49, 2.36)
Mean Arterial Pressure (mmHg)	367	0.57 (−0.89, 2.03)	−0.32 (−2.07, 1.43)	−0.77 (−3.65, 2.11)	0.65 (−1.14, 2.43)	−0.11 (−2.87, 2.66)
Pulse Pressure (mmHg)	367	0.43 (−1.02, 1.88)	−0.04 (−1.77, 1.69)	0.33 (−2.51, 3.16)	0.44 (−1.33, 2.20)	−0.59 (−3.31, 2.13)
Glucose (mg/dL)	374	1.14 (−2.51, 4.79)	−0.30 (−4.27, 3.67)	−0.96 (−5.89, 3.98)	−0.83 (−4.72, 3.05)	−1.08 (−5.80, 3.64)
HbA1c (%)	374	0.10 (−0.01, 0.22)	0.05 (−0.08, 0.18)	0.00 (−0.17, 0.17)	−0.03 (−0.16, 0.10)	−0.01 (−0.17, 0.15)
HDL-C (mg/dL)	393	−0.23 (−1.96, 1.49)	0.76 (−1.30, 2.82)	0.97 (−2.36, 4.31)	1.38 (−0.66, 3.42)	−1.72 (−4.86, 1.43)
Triglycerides^d^ (mg/dL)	393	0.02 (−0.01, 0.05)	0.01 (−0.03, 0.05)	0.01 (−0.05, 0.07)	−0.03 (−0.07, 0.00)	0.08 (0.02, 0.13)*
hs-CRP^d^ (mg/dL)	453	0.04 (−0.03, 0.11)	−0.02 (−0.10, 0.07)	−0.05 (−0.18, 0.09)	0.11 (0.03, 0.19)*	−0.03 (−0.16, 0.09)
IL-6^d^ (pg/mL)	454	0.03 (−0.01, 0.07)	0.00 (−0.05, 0.05)	0.00 (−0.08, 0.09)	0.08 (0.03, 0.14)*	0.00 (−0.08, 0.08)

Abbreviations: *p,p*′-DDT, *p,p*′-dichlorodiphenyltrichloroethane; *p,p*′-DDE, *p,p*′-dichlorodiphenyldichloroethylene; HCB, hexachlorobenzene; β-HCH, β-hexachlorocyclohexane; HbA1c, glycated hemoglobin; HDL-C, high-density lipoprotein cholesterol; hs-CRP, high sensitivity C-reactive protein; IL-6, interleukin 6.

Notes: Results expressed as beta coefficient and 95% credible interval (CrI) per 10-fold increase in OC pesticide exposure.

a Adjusted for individual OC pesticides, age at outcome assessment (2022–2024), poverty status at exposure assessment (2009–2011), and years lived in the U.S. at exposure assessment (2009–2011).

b Adjusted for multiple exposures without DDT due to high correlation.

c Blood pressure measures excluded 101 participants who report current hypertension medication; Glucose and HbA1c exclude 79 participants who report current medication for diabetes; HDL-C and Triglycerides exclude 60 participants who report current cholesterol lowering medication.

d log transformed.

* 95% Credible Interval does not include the null hypothesis (0.0).

**Table 5:** Adjusted^a^ associations between OC pesticide exposure (log_10_) and binary cardiometabolic and Inflammatory outcomes using semi-Bayesian Hierarchical Models, CHAMACOS Maternal Cognition Study, Salinas, CA 2022–2024.

Outcome	n (%)	Exposure (ng/g lipid) *p,p*′-DDT RR (95 % CrI)	*p,p*′-DDE^b^ RR (95 % CrI)	HCB RR (95 % CrI)	β-HCH RR (95 % CrI)	Trans-nonachlor RR (95 % CrI)
Obesity (≥30 kg/m^2^)	305 (65.2)	1.11 (1.01, 1.23)*	1.03 (0.91, 1.18)	1.06 (0.85, 1.33)	1.16 (1.01, 1.33)*	0.90 (0.72, 1.13)
Waist circumference (≥88 cm)	407 (87.0)	1.04 (0.98, 1.10)	1.02 (0.94, 1.10)	1.05 (0.92, 1.21)	1.06 (0.99, 1.14)	0.97 (0.85, 1.10)
Triglycerides (≥150 mg/dL or drug tx)	202 (44.6)	1.06 (0.90, 1.25)	0.98 (0.79, 1.22)	1.04 (0.74, 1.47)	0.87 (0.71, 1.05)	1.49 (1.11, 2.00)*
HDL-C (<50 mg/dL or drug tx)	265 (58.5)	1.04 (0.92, 1.18)	1.01 (0.85, 1.18)	0.81 (0.61, 1.08)	0.99 (0.85, 1.16)	1.22 (0.95, 1.57)
Increased Blood Pressure^c^	180 (38.5)	0.94 (0.77, 1.14)	0.86 (0.68, 1.09)	0.91 (0.62, 1.33)	1.16 (0.92, 1.46)	1.12 (0.78, 1.61)
Glucose (≥100 mg/dL or drug tx)	159 (35.2)	1.09 (0.91, 1.30)	0.89 (0.69, 1.14)	0.67 (0.44, 1.04)	1.10 (0.88, 1.39)	1.18 (0.82, 1.72)
Metabolic Syndrome^d^	220 (48.7)	0.98 (0.85, 1.13)	0.85 (0.70, 1.03)	0.87 (0.63, 1.19)	1.06 (0.89, 1.26)	1.30 (0.99, 1.71)
Diabetes^e^	161 (34.0)	1.16 (0.98, 1.39)	0.99 (0.78, 1.25)	0.94 (0.64, 1.40)	0.98 (0.78, 1.23)	0.83 (0.56, 1.23)
Elevated hs-CRP (>3.0 mg/dL)	212 (46.8)	1.11 (0.96, 1.27)	1.00 (0.82, 1.21)	0.80 (0.57, 1.14)	1.18 (0.97, 1.44)	1.00 (0.73, 1.37)

Abbreviations: *p,p*′-DDT, *p,p*′-dichlorodiphenyltrichloroethane; *p,p*′-DDE, *p,p*′-dichlorodiphenyldichloroethylene; HCB, hexachlorobenzene; β-HCH, β-hexachlorocyclohexane; HbA1c, glycated hemoglobin; HDL-C, high-density lipoprotein cholesterol; hs-CRP, high sensitivity C-reactive protein.

Notes: Results expressed as Relative Risk (RR) and 95% credible interval (CrI) per 10-fold increase in OC pesticide exposure.

a Adjusted for individual OC pesticides, age at outcome assessment (2022–2024), poverty status at exposure assessment (2009–2011), and years lived in the U.S. at exposure assessment (2009–2011).

b Adjusted for multiple exposure without DDT due to high correlation.

c Systolic blood pressure ≥ 130 mm Hg or diastolic blood pressure ≥ 85 mm Hg or drug treatment for high blood pressure.

d Metabolic Syndrome presence of ≥ 3 of: (1) waist circumference ≥ 88 cm; (2) serum triglycerides ≥ 150 mg/dL or drug treatment to lower cholesterol; (3) serum HDL-C < 50 mg/dL or drug treatment to lower cholesterol; (4) systolic blood pressure ≥ 130 mm Hg or diastolic blood pressure ≥ 85 mm Hg or drug treatment for high blood pressure;(5) serum glucose ≥ 100 mg/dL or drug treatment for diabetes.

e Diabetes (self-report of drug treatment for diabetes, HbA1c ≥ 6.5 %, glucose ≥ 126 mg/dL).

* 95% Credible Interval does not include the null hypothesis (0.0).

## Data Availability

The authors do not have permission to share data.
