# Supplementary material for: Persistent organochlorine pesticides and cardiometabolic outcomes among middle-aged Latina women in a California agricultural community: The CHAMACOS Maternal Cognition Study
Source: Environ Int. Author manuscript; Available in PMC 2026 Feb 1. (PMC12686108; doi:10.1016/j.envint.2025.109302)

Supplementary Table 1. Spearman correlation matrix for persistent organochlorine pesticide concentrations (N=466)

|  | *p,p'*-DDT | *p,p'*-DDE | HCB | β-HCH | Trans-nonachlor |
| --- | --- | --- | --- | --- | --- |
| *p,p'*-DDT | 1.000 | ------ | ------ | ------ | ------ |
| *p,p'*-DDE | 0.824 | 1.000 | ------ | ------ | ------ |
| HCB | 0.245 | 0.366 | 1.000 | ------ | ------ |
| β-HCH | 0.225 | 0.264 | 0.372 | 1.000 | ------ |
| Trans-nonachlor | 0.308 | 0.431 | 0.523 | 0.466 | 1.000 |

Abbreviations: *p,p*′-DDT, *p,p*′-dichlorodiphenyltrichloroethane; *p,p*′-DDE, *p,p*′-dichlorodiphenyldichloroethylene; HCB, hexachlorobenzene; β-HCH, β-hexachlorocyclohexane

Supplementary Table 2. Sensitivity Analysis: Adjusted^a^ associations between OC pesticide exposure (log_10_) and continuous cardiometabolic and inflammatory outcomes using linear semi-Bayesian Hierarchical Models excluding 66 participants with non-fasting blood draw, CHAMACOS Maternal Cognition Study, Salinas, CA 2022-2024

|  |  | *p,p'*-DDT | *p,p'*-DDE^b^ | HCB | β-HCH | Trans-nonachlor |
| --- | --- | --- | --- | --- | --- | --- |
| Outcome | n^c^ | β (95% CrI) | β (95% CrI) | β (95% CrI) | β (95% CrI) | β (95% CrI) |
|  |  |  |  |  |  |  |
| Glucose (mg/dL) | 316 | 0.12 (-3.84, 4.07) | -1.28 (-5.48, 2.93) | -1.16 (-6.16, 3.84) | -1.30 (-5.43, 2.83) | -1.45 (-6.35, 3.45) |
| HbA1c (%) | 316 | 0.10 (-0.03, 0.23) | 0.04 (-0.11, 0.18) | 0.02 (-0.16, 0.19) | -0.02 (-0.16, 0.12) | 0.00 (-0.17, 0.18) |
| HDL-C (mg/dL) | 332 | -0.18 (-2.02, 1.65) | 0.02 (-0.07, 0.10) | 1.43 (-1.96, 4.83) | 1.55 (-0.61, 3.71) | -1.12 (-4.51, 2.26) |
| Triglycerides^d^ (mg/dL) | 332 | 0.02 (-0.01, 0.05) | 0.01 (-0.03, 0.04) | 0.00 (-0.06, 0.06) | -0.04 (-0.07, 0.00) | 0.05 (-0.01, 0.11) |
| hs-CRP^d^ (mg/dL) | 387 | 0.03 (-0.04, 0.10) | -0.03 (-0.12, 0.05) | -0.03 (-0.17, 0.10) | 0.08 ( 0.00, 0.17)* | -0.06 (-0.19, 0.08) |
| IL-6^d^ (pg/mL) | 388 | 0.01 (-0.03, 0.06) | -0.02 (-0.08, 0.03) | 0.00 (-0.09, 0.08) | 0.07 ( 0.02, 0.12)* | -0.05 (-0.13, 0.04) |

Abbreviations: *p,p*′-DDT, *p,p*′-dichlorodiphenyltrichloroethane; *p,p*′-DDE, *p,p*′-dichlorodiphenyldichloroethylene; HCB, hexachlorobenzene; β-HCH, β-hexachlorocyclohexane; HbA1c, glycated hemoglobin; HDL-C, high-density lipoprotein cholesterol; hs-CRP, high sensitivity C-reactive protein; IL-6, interleukin 6

Notes: Results expressed as beta coefficient and 95% credible interval (CrI) per 10-fold increase in OC pesticide exposure.

^a^ Adjusted for individual OC pesticides, age at outcome assessment (2022-2024), poverty status at exposure assessment (2009-2011), and years lived in the U.S. at exposure assessment (2009-2011)

^b^ Adjusted for multiple exposure without DDT due to high correlation

^c^ Blood pressure measures excluded 101 participants who report current hypertension medication; Glucose and HbA1c exclude 79 participants who report current medication for diabetes; HDL-C and Triglycerides exclude 60 participants who report current cholesterol lowering medication

^d^ log transformed

* 95% Credible Interval does not include the null hypothesis (0.0)

Supplementary Table 3. Sensitivity Analysis: Adjusted^a^ associations between OC pesticide exposure (log_10_) and binary cardiometabolic and inflammatory outcomes using semi-Bayesian Hierarchical Models excluding 66 participants with non-fasting blood draw, CHAMACOS Maternal Cognition Study, Salinas, CA 2022-2024

|  |  | Exposure (ng/g lipid) | | | | |
| --- | --- | --- | --- | --- | --- | --- |
|  |  | *p,p'*-DDT | *p,p'*-DDE^b^ | HCB | β-HCH | Trans-nonachlor |
| Outcome | n (%) | RR (95% CrI) | RR (95% CrI) | RR (95% CrI) | RR (95% CrI) | RR (95% CrI) |
|  |  |  |  |  |  |  |
| Triglycerides (≥150mg/dL or tx) | 166 (43.0) | 0.99 ( 0.81, 1.21) | 0.90 ( 0.70, 1.15) | 1.00 ( 0.68, 1.46) | 0.83 ( 0.67, 1.02) | 1.46 ( 1.01, 2.11)* |
| HDL-C (<50mg/dL or tx) | 226 (58.6) | 1.01 ( 0.88, 1.16) | 0.95 ( 0.79, 1.13) | 0.78 ( 0.58, 1.06) | 1.00 ( 0.84, 1.18) | 1.28 ( 0.96, 1.72) |
|  |  |  |  |  |  |  |
| Glucose (≥100mg/dL or drug tx) | 132 (34.2) | 1.07 ( 0.88, 1.31) | 0.85 ( 0.64, 1.13) | 0.69 ( 0.44, 1.09) | 1.07 ( 0.83, 1.38) | 1.06 ( 0.68, 1.63) |
|  |  |  |  |  |  |  |
| Diabetes^e^ | 158 (33.8) | 1.10 ( 0.91, 1.33) | 0.91 ( 0.70, 1.17) | 0.96 ( 0.65, 1.44) | 0.96 ( 0.75, 1.21) | 0.91 ( 0.59, 1.39) |
|  |  |  |  |  |  |  |
| Elevated hs-CRP (>3.0 mg/dL) | 179 (46.2) | 1.11 ( 0.95, 1.29) | 0.97 ( 0.78, 1.20) | 0.81 ( 0.56, 1.17) | 1.12 ( 0.91, 1.38) | 1.03 ( 0.72, 1.47) |

Abbreviations: *p,p*′-DDT, *p,p*′-dichlorodiphenyltrichloroethane; *p,p*′-DDE, *p,p*′-dichlorodiphenyldichloroethylene; HCB, hexachlorobenzene; β-HCH, β-hexachlorocyclohexane; HbA1c, glycated hemoglobin; HDL-C, high-density lipoprotein cholesterol; hs-CRP, high sensitivity C-reactive protein

Notes: Results expressed as Relative Risk (RR) and 95% credible interval (CrI) per 10-fold increase in OC pesticide exposure.

^a^ Adjusted for individual OC pesticides, age at outcome assessment (2022-2024), poverty status at exposure assessment (2009-2011), and years lived in the U.S. at exposure assessment (2009-2011)

^b^ Adjusted for multiple exposure without DDT due to high correlation

^c^ Systolic blood pressure ≥130 mm Hg or diastolic blood pressure ≥ 85 mm Hg or drug treatment for high blood pressure

^d^ Metabolic Syndrome presence of ≥3 of: (1) waist circumference ≥88 cm; (2) serum triglycerides ≥150 mg/dL; (3) serum HDL-C<50 mg/dL; (4) systolic blood pressure ≥130 mm Hg or diastolic blood pressure ≥ 85 mm Hg or drug treatment for high blood pressure;(5) serum glucose ≥100 mg/dL or drug treatment for diabetes.

**^e^** Diabetes (self-report of drug treatment for diabetes, HbA1c ≥ 6.5%, glucose≥126 mg/dL)

* 95% Credible Interval does not include the null hypothesis (0.0)

Supplementary Table 4. Sensitivity Analysis: Adjusted^a^ associations between OC pesticide exposure (log_10_) and continuous cardiometabolic and inflammatory outcomes using linear semi-Bayesian Hierarchical Models excluding outliers (>±3SD), CHAMACOS Maternal Cognition Study, Salinas, CA 2022-2024

|  |  | *p,p'*-DDT | *p,p'*-DDE^b^ | HCB | β-HCH | Trans-nonachlor |
| --- | --- | --- | --- | --- | --- | --- |
| Outcome | n^c^ | β (95% CrI) | β (95% CrI) | β (95% CrI) | β (95% CrI) | β (95% CrI) |
|  |  |  |  |  |  |  |
| Body Mass Index (kg/m^2^) | 460 | 1.07 ( 0.21, 1.93)* | 0.48 (-0.56, 1.52) | -0.47 (-2.15, 1.21) | 1.46 ( 0.44, 2.49)* | -0.06 (-1.68, 1.57) |
| Waist Circumference (cm) | 462 | 3.04 ( 1.07, 5.01)* | 1.62 (-0.77, 4.01) | 0.02 (-3.83, 3.88) | 3.58 ( 1.22, 5.94)* | 0.94 (-2.77, 4.65) |
|  |  |  |  |  |  |  |
| Systolic Blood Pressure (mmHg) | 367 | 0.74 (-1.32, 2.79) | -0.39 (-2.85, 2.06) | -0.46 (-4.47, 3.56) | 0.85 (-1.66, 3.36) | -0.65 (-4.50, 3.20) |
| Diastolic Blood Pressure (mmHg) | 367 | 0.32 (-0.94, 1.57) | -0.36 (-1.87, 1.15) | -0.86 (-3.38, 1.67) | 0.44 (-1.10, 1.98) | -0.07 (-2.49, 2.36) |
| Mean Arterial Pressure (mmHg) | 367 | 0.57 (-0.89, 2.03) | -0.32 (-2.07, 1.43) | -0.77 (-3.65, 2.11) | 0.65 (-1.14, 2.43) | -0.11 (-2.87, 2.66) |
| Pulse Pressure (mmHg) | 365 | 0.48 (-0.95, 1.90) | 0.04 (-1.66, 1.75) | 0.41 (-2.40, 3.21) | 0.43 (-1.31, 2.17) | -0.39 (-3.08, 2.31) |
|  |  |  |  |  |  |  |
| Glucose (mg/dL) | 369 | 2.36 (-0.04, 4.76) | 0.89 (-1.85, 3.62) | -2.02 (-5.86, 1.82) | -0.17 (-2.85, 2.50) | -0.61 (-4.25, 3.03) |
| HbA1c (%) | 369 | 0.10 ( 0.02, 0.17)* | 0.04 (-0.05, 0.13) | -0.07 (-0.21, 0.06) | 0.01 (-0.07, 0.10) | 0.00 (-0.13, 0.13) |
| HDL-C (mg/dL) | 391 | -0.05 (-1.75, 1.64) | 0.70 (-1.32, 2.73) | 0.78 (-2.52, 4.07) | 1.49 (-0.52, 3.49) | -1.55 (-4.66, 1.56) |
| Triglycerides^d^ (mg/dL) | 389 | 0.02 (-0.01, 0.05) | 0.02 (-0.02, 0.05) | 0.02 (-0.03, 0.08) | -0.03 (-0.06, 0.01) | 0.06 ( 0.01, 0.11)* |
| hs-CRP^d^ (mg/dL) | 451 | 0.04 (-0.03, 0.11) | -0.02 (-0.10, 0.06) | -0.06 (-0.19, 0.07) | 0.10 ( 0.02, 0.18)* | -0.02 (-0.15, 0.10) |
| IL-6^d^ (pg/mL) | 447 | 0.03 (-0.02, 0.07) | 0.00 (-0.05, 0.05) | 0.01 (-0.07, 0.09) | 0.07 ( 0.02, 0.12)* | 0.00 (-0.08, 0.07) |

Abbreviations: *p,p*′-DDT, *p,p*′-dichlorodiphenyltrichloroethane; *p,p*′-DDE, *p,p*′-dichlorodiphenyldichloroethylene; HCB, hexachlorobenzene; β-HCH, β-hexachlorocyclohexane; HbA1c, glycated hemoglobin; HDL-C, high-density lipoprotein cholesterol; hs-CRP, high sensitivity C-reactive protein; IL-6, interleukin 6

Notes: Results expressed as beta coefficient and 95% credible interval (CrI) per 10-fold increase in OC pesticide exposure.

^a^ Adjusted for individual OC pesticides, age at outcome assessment (2022-2024), poverty status at exposure assessment (2009-2011), and years lived in the U.S. at exposure assessment (2009-2011)

^b^ Adjusted for multiple exposure without DDT due to high correlation

^c^ Blood pressure measures excluded 101 participants who report current hypertension medication; Glucose and HbA1c exclude 79 participants who report current medication for diabetes; HDL-C and Triglycerides exclude 60 participants who report current cholesterol lowering medication

^d^ log transformed

* 95% Credible Interval does not include the null hypothesis (0.0)Supplementary Table 5. Comparison of estimates for HCB, β-HCH, and trans-nonachlor with DDT versus with DDE in the model. Adjusted^a^ associations between OC pesticide exposure (log_10_) and continuous cardiometabolic and inflammatory outcomes using linear semi-Bayesian Hierarchical Models, CHAMACOS Maternal Cognition Study, Salinas, CA 2022-2024

|  |  | DDT or DDE | HCB | β-HCH | Trans-nonachlor |
| --- | --- | --- | --- | --- | --- |
| Outcome | n^b^ | In Model | β (95% CrI) | β (95% CrI) | β (95% CrI) |
|  |  |  |  |  |  |
| Body Mass Index (kg/m^2^) | 468 | with DDT | -0.24 (-2.03, 1.56) | 1.56 ( 0.45, 2.67)* | -0.25 (-1.98, 1.48) |
|  |  | with DDE | -0.30 (-2.10, 1.51) | 1.59 ( 0.48, 2.71)* | -0.16 (-1.91, 1.59) |
| Waist Circumference (cm) | 468 | with DDT | 0.42 (-3.62, 4.46) | 3.74 ( 1.24, 6.23)* | 0.65 (-3.24, 4.54) |
|  |  | with DDE | 0.30 (-3.77, 4.36) | 3.82 ( 1.31, 6.33)* | 0.86 (-3.08, 4.79) |
| Systolic Blood Pressure (mmHg) | 367 | with DDT | -0.46 (-4.47, 3.56) | 0.85 (-1.66, 3.36) | -0.65 (-4.50, 3.20) |
|  |  | with DDE | -0.41 (-4.43, 3.61) | 0.91 (-1.60, 3.43) | -0.35 (-4.24, 3.54) |
| Diastolic Blood Pressure (mmHg) | 367 | with DDT | -0.86 (-3.38, 1.67) | 0.44 (-1.10, 1.98) | -0.07 (-2.49, 2.36) |
|  |  | with DDE | -0.81 (-3.34, 1.73) | 0.48 (-1.07, 2.03) | 0.14 (-2.31, 2.59) |
| Mean Arterial Pressure (mmHg) | 367 | with DDT | -0.77 (-3.65, 2.11) | 0.65 (-1.14, 2.43) | -0.11 (-2.87, 2.66) |
|  |  | with DDE | -0.72 (-3.61, 2.17) | 0.70 (-1.09, 2.49) | 0.14 (-2.65, 2.94) |
| Pulse Pressure (mmHg) | 367 | with DDT | 0.33 (-2.51, 3.16) | 0.44 (-1.33, 2.20) | -0.59 (-3.31, 2.13) |
|  |  | with DDE | -0.72 (-3.61, 2.17) | 0.70 (-1.09, 2.49) | 0.14 (-2.65, 2.94) |
| Glucose (mg/dL) | 374 | with DDT | -0.96 (-5.89, 3.98) | -0.83 (-4.72, 3.05) | -1.08 (-5.80, 3.64) |
|  |  | with DDE | -1.34 (-6.26, 3.58) | -1.04 (-4.92, 2.83) | -1.37 (-6.08, 3.35) |
| HbA1c (%) | 374 | with DDT | 0.00 (-0.17, 0.17) | -0.03 (-0.16, 0.10) | -0.01 (-0.17, 0.15) |
|  |  | with DDE | -0.01 (-0.18, 0.15) | -0.03 (-0.16, 0.09) | -0.02 (-0.18, 0.14) |
| HDL-C (mg/dL) | 393 | with DDT | 0.97 (-2.36, 4.31) | 1.38 (-0.66, 3.42) | -1.72 (-4.86, 1.43) |
|  |  | with DDE | 0.88 (-2.46, 4.23) | 1.32 (-0.73, 3.36) | -1.95 (-5.12, 1.22) |
| Triglycerides^c^ (mg/dL) | 393 | with DDT | 0.01 (-0.05, 0.07) | -0.03 (-0.07, 0.00) | 0.08 ( 0.02, 0.13)* |
|  |  | with DDE | 0.01 (-0.05, 0.07) | -0.03 (-0.07, 0.00) | 0.08 ( 0.02, 0.13)* |
| hs-CRP^c^ (mg/dL) | 453 | with DDT | -0.05 (-0.18, 0.09) | 0.11 ( 0.03, 0.19)* | -0.03 (-0.16, 0.09) |
|  |  | with DDE | -0.04 (-0.18, 0.09) | 0.11 ( 0.03, 0.19)* | -0.02 (-0.15, 0.11) |
| IL-6^c^ (pg/mL) | 454 | with DDT | 0.00 (-0.08, 0.09) | 0.08 ( 0.03, 0.14)* | 0.00 (-0.08, 0.08) |
|  |  | with DDE | 0.00 (-0.08, 0.09) | 0.09 ( 0.03, 0.14)* | 0.00 (-0.08, 0.08) |

Abbreviations: *p,p*′-DDT, *p,p*′-dichlorodiphenyltrichloroethane; *p,p*′-DDE, *p,p*′-dichlorodiphenyldichloroethylene; HCB, hexachlorobenzene; β-HCH, β-hexachlorocyclohexane; HbA1c, glycated hemoglobin; HDL-C, high-density lipoprotein cholesterol; hs-CRP, high sensitivity C-reactive protein; IL-6, interleukin 6

Notes: Results expressed as beta coefficient and 95% credible interval (CrI) per 10-fold increase in OC pesticide exposure.

^a^ Adjusted for individual OC pesticides, age at outcome assessment (2022-2024), poverty status at exposure assessment (2009-2011), and years lived in the U.S. at exposure assessment (2009-2011)

^b^ Blood pressure measures excluded 101 participants who report current hypertension medication; Glucose and HbA1c exclude 79 participants who report current medication for diabetes; HDL-C and Triglycerides exclude 60 participants who report current cholesterol lowering medication

^c^ log transformed

* 95% Credible Interval does not include the null hypothesis (0.0)

Supplementary Table 6. Comparison of estimates for HCB, β-HCH, and trans-nonachlor with DDT versus with DDE in the model. Adjusted^a^ associations between OC pesticide exposure (log_10_) and binary cardiometabolic and inflammatory outcomes using semi-Bayesian Hierarchical Models, CHAMACOS Maternal Cognition Study, Salinas, CA 2022-2024

|  |  |  | Exposure (ng/g lipid) | | |
| --- | --- | --- | --- | --- | --- |
|  |  | DDT or DDE | HCB | β-HCH | Trans-nonachlor |
| Outcome | n (%) | in Model | RR (95% CrI) | RR (95% CrI) | RR (95% CrI) |
|  |  |  |  |  |  |
| Obesity (≥30 kg/m^2^) | 305 (65.2) | with DDT | 1.06 ( 0.85, 1.33) | 1.16 ( 1.01, 1.33)* | 0.90 ( 0.72, 1.13) |
|  |  | with DDE | 1.06 ( 0.84, 1.33) | 1.17 ( 1.02, 1.34)* | 0.92 ( 0.73, 1.16) |
|  |  |  |  |  |  |
| Waist circumference (≥88 cm) | 407 (87.0) | with DDT | 1.05 ( 0.92, 1.21) | 1.06 ( 0.99, 1.14) | 0.97 ( 0.85, 1.10) |
|  |  | with DDE | 1.05 ( 0.91, 1.21) | 1.06 ( 0.99, 1.14) | 0.97 ( 0.85, 1.12) |
| Triglycerides (≥150mg/dL or drug tx) | 202 (44.6) | with DDT | 1.04 ( 0.74, 1.47) | 0.87 ( 0.71, 1.05) | 1.49 ( 1.11, 2.00)* |
|  |  | with DDE | 1.04 ( 0.74, 1.48) | 0.87 ( 0.72, 1.05) | 1.53 ( 1.13, 2.07)* |
| HDL-C (<50mg/dL or drug tx) | 265 (58.5) | with DDT | 0.81 ( 0.61, 1.08) | 0.99 ( 0.85, 1.16) | 1.22 ( 0.95, 1.57) |
|  |  | with DDE | 0.81 ( 0.61, 1.09) | 0.99 ( 0.85, 1.17) | 1.24 ( 0.96, 1.59) |
| Increased Blood Pressure^b^ | 180 (38.5) | with DDT | 0.91 ( 0.62, 1.33) | 1.16 ( 0.92, 1.46) | 1.12 ( 0.78, 1.61) |
|  |  | with DDE | 0.93 ( 0.64, 1.36) | 1.17 ( 0.93, 1.47) | 1.16 ( 0.80, 1.68) |
| Glucose (≥100mg/dL or drug tx) | 159 (35.2) | with DDT | 0.67 ( 0.44, 1.04) | 1.10 ( 0.88, 1.39) | 1.18 ( 0.82, 1.72) |
|  |  | with DDE | 0.68 ( 0.44, 1.05) | 1.12 ( 0.89, 1.42) | 1.25 ( 0.85, 1.84) |
| Metabolic Syndrome^c^ | 220 (48.7) | with DDT | 0.87 ( 0.63, 1.19) | 1.06 ( 0.89, 1.26) | 1.30 ( 0.99, 1.71) |
|  |  | with DDE | 0.89 ( 0.65, 1.22) | 1.07 ( 0.90, 1.27) | 1.37 ( 1.04, 1.82)* |
|  |  |  |  |  |  |
| Diabetes^d^ | 161 (34.0) | with DDT | 0.94 ( 0.64, 1.40) | 0.98 ( 0.78, 1.23) | 0.83 ( 0.56, 1.23) |
|  |  | with DDE | 0.95 ( 0.63, 1.41) | 0.99 ( 0.78, 1.25) | 0.87 ( 0.58, 1.30) |
|  |  |  |  |  |  |
| Elevated hs-CRP (>3.0 mg/dL) | 212 (46.8) | with DDT | 0.80 ( 0.57, 1.14) | 1.18 ( 0.97, 1.44) | 1.00 ( 0.73, 1.37) |
|  |  | with DDE | 0.81 ( 0.57, 1.15) | 1.19 ( 0.98, 1.46) | 1.02 ( 0.74, 1.41) |

Abbreviations: *p,p*′-DDT, *p,p*′-dichlorodiphenyltrichloroethane; *p,p*′-DDE, *p,p*′-dichlorodiphenyldichloroethylene; HCB, hexachlorobenzene; β-HCH, β-hexachlorocyclohexane; HbA1c, glycated hemoglobin; HDL-C, high-density lipoprotein cholesterol; hs-CRP, high sensitivity C-reactive protein

Notes: Results expressed as Relative Risk (RR) and 95% credible interval (CrI) per 10-fold increase in OC pesticide exposure.

^a^ Adjusted for individual OC pesticides, age at outcome assessment (2022-2024), poverty status at exposure assessment (2009-2011), and years lived in the U.S. at exposure assessment (2009-2011)^b^ Systolic blood pressure ≥130 mm Hg or diastolic blood pressure ≥ 85 mm Hg or drug treatment for high blood pressure

^c^ Metabolic Syndrome presence of ≥3 of: (1) waist circumference ≥88 cm; (2) serum triglycerides ≥150 mg/dL or drug treatment to lower cholesterol; (3) serum HDL-C<50 mg/dL or drug treatment to lower cholesterol; (4) systolic blood pressure ≥130 mm Hg or diastolic blood pressure ≥ 85 mm Hg or drug treatment for high blood pressure;(5) serum glucose ≥100 mg/dL or drug treatment for diabetes.

**^d^** Diabetes (self-report of drug treatment for diabetes, HbA1c ≥ 6.5%, glucose≥126 mg/dL)

* 95% Credible Interval does not include the null hypothesis (0.0)

Supplementary Table 7 - Sensitivity Analysis: Adjusted^a^ associations between individual OC pesticide exposure (log_10_) and continuous cardiometabolic and inflammatory outcomes using multivariable linear regression models, CHAMACOS Maternal Cognition Study, Salinas, CA 2022-2024

|  |  | *p,p'*-DDT | *p,p'*-DDE | HCB | β-HCH | Trans-nonachlor |
| --- | --- | --- | --- | --- | --- | --- |
| Outcome | n^c^ | β (95% CI) | β (95% CI) | β (95% CI) | β (95% CI) | β (95% CI) |
|  |  |  |  |  |  |  |
| Body Mass Index (kg/m^2^) | 468 | 1.53 (0.55, 2.52)* | 0.88 (-0.32, 2.08) | 0.19 (-2.01, 2.39) | 1.82 (0.69, 2.96)* | 0.81 (-1.17, 2.79) |
| Waist Circumference (cm) | 468 | 3.48 (1.26, 5.71)* | 2.18 (-0.51, 4.88) | 2.31 (-2.64, 7.26) | 4.71 (2.15, 7.26)* | 3.88 (-0.57, 8.32) |
|  |  |  |  |  |  |  |
| Systolic Blood Pressure (mmHg) | 367 | 0.86 (-1.27, 2.98) | -0.35 (-2.89, 2.18) | -0.54 (-5.30, 4.22) | 0.86 (-1.69, 3.41) | -0.35 (-4.60, 3.91) |
| Diastolic Blood Pressure (mmHg) | 367 | 0.37 (-0.91, 1.65) | -0.35 (-1.88, 1.17) | -1.01 (-3.87, 1.85) | 0.41 (-1.13, 1.94) | 0.02 (-2.53, 2.58) |
| Mean Arterial Pressure (mmHg) | 367 | 0.68 (-0.82, 2.18) | -0.26 (-2.05, 1.53) | -0.85 (-4.21, 2.50) | 0.68 (-1.11, 2.48) | 0.19 (-2.81, 3.18) |
| Pulse Pressure (mmHg) | 367 | 0.49 (-1.01, 1.98) | 0.00 (-1.78, 1.78) | 0.47 (-2.87, 3.81) | 0.45 (-1.33, 2.24) | -0.37 (-3.35, 2.61) |
|  |  |  |  |  |  |  |
| Glucose (mg/dL) | 374 | 2.53 (-2.20, 7.26) | 0.11 (-5.55, 5.77) | -4.35 (-14.85, 6.16) | -1.68 (-7.16, 3.79) | -4.20 (-13.45, 5.06) |
| HbA1c (%) | 374 | 0.17 (0.02, 0.32)* | 0.09 (-0.08, 0.27) | -0.05 (-0.38, 0.28) | -0.07 (-0.24, 0.10) | -0.08 (-0.37, 0.21) |
| HDL-C (mg/dL) | 393 | -0.23 (-2.03, 1.57) | 0.85 (-1.30, 3.00) | 1.32 (-2.67, 5.30) | 1.26 (-0.81, 3.33) | -1.36 (-4.82, 2.11) |
| Triglycerides^b^ (mg/dL) | 393 | 0.02 (-0.01, 0.06) | 0.02 (-0.02, 0.06) | 0.03 (-0.04, 0.11) | -0.02 (-0.06, 0.02) | 0.09 (0.03, 0.15)* |
| hs-CRP^b^ (mg/dL) | 453 | 0.05 (-0.02, 0.12) | -0.01 (-0.09, 0.08) | -0.03 (-0.18, 0.12) | 0.11 (0.03, 0.19)* | 0.02 (-0.11, 0.16) |
| IL-6^b^ (pg/mL) | 454 | 0.04 (-0.01, 0.09) | 0.01 (-0.04, 0.07) | 0.03 (-0.07, 0.14) | 0.10 (0.05, 0.15)* | 0.05 (-0.04, 0.15) |

Abbreviations: *p,p*′-DDT, *p,p*′-dichlorodiphenyltrichloroethane; *p,p*′-DDE, *p,p*′-dichlorodiphenyldichloroethylene; HCB, hexachlorobenzene; β-HCH, β-hexachlorocyclohexane; HbA1c, glycated hemoglobin; HDL-C, high-density lipoprotein cholesterol; hs-CRP, high sensitivity C-reactive protein;IL-6, interleukin 6

Notes: Results expressed as beta coefficient and 95% confidence interval (CI) per 10-fold increase in OC pesticide exposure.

^a^ Adjusted for individual OC pesticides, age at outcome assessment (2022-2024), poverty status at exposure assessment (2009-2011), and years lived in the U.S. at exposure assessment (2009-2011)

^b^ log transformed

^c^ Blood pressure measures excluded 101 participants who report current hypertension medication; Glucose and HbA1c exclude 79 participants who report current medication for diabetes; HDL-C and Triglycerides exclude 60 participants who report current cholesterol lowering medication

* 95% Confidence Interval does not include the null hypothesis (0.0)

Supplementary Table 8 - Sensitivity Analysis: Adjusted^a^ associations between individual OC pesticide exposure (log_10_) and binary cardiometabolic and inflammatory outcomes using multivariable Poisson regression models, CHAMACOS Maternal Cognition Study, Salinas, CA 2022-2024

|  |  | Exposure (ng/g lipid) | | | | |
| --- | --- | --- | --- | --- | --- | --- |
|  |  | *p,p'*-DDT | *p,p'*-DDE | HCB | β-HCH | Trans-nonachlor |
| Outcome | n (%) | RR (95% CI) | RR (95% CI) | RR (95% CI) | RR (95% CI) | RR (95% CI) |
|  |  |  |  |  |  |  |
| Obesity (≥30 kg/m^2^) | 305 (65.2) | 1.13 (1.02, 1.24)* | 1.06 (0.93, 1.20) | 1.12 (0.91, 1.38) | 1.17 (1.03, 1.32)* | 1.06 (0.86, 1.30) |
| Waist circumference (≥88 cm) | 407 (87.0) | 1.04 (0.99, 1.10) | 1.03 (0.96, 1.10) | 1.08 (0.95, 1.23) | 1.07 (1.01, 1.14)* | 1.05 (0.94, 1.16) |
| Triglycerides (≥150mg/dL or drug tx) | 202 (44.6) | 1.09 (0.93, 1.28) | 1.04 (0.85, 1.27) | 1.14 (0.80, 1.63) | 0.95 (0.78, 1.15) | 1.46 (1.10, 1.94)* |
| HDL-C (<50mg/dL or drug tx) | 265 (58.5) | 1.05 (0.93, 1.19) | 1.02 (0.87, 1.19) | 0.87 (0.66, 1.15) | 1.02 (0.88, 1.18) | 1.17 (0.93, 1.47) |
| Increased Blood Pressure^c^ | 180 (38.5) | 0.96 (0.80, 1.16) | 0.89 (0.72, 1.12) | 0.98 (0.67, 1.43) | 1.18 (0.95, 1.46) | 1.19 (0.83, 1.71) |
| Glucose (≥100mg/dL or drug tx) | 159 (35.2) | 1.10 (0.91, 1.34) | 0.90 (0.70, 1.17) | 0.65 (0.41, 1.03) | 1.11 (0.89, 1.40) | 1.21 (0.81, 1.81) |
| Metabolic Syndrome^d^ | 220 (48.7) | 1.01 (0.88, 1.16) | 0.90 (0.76, 1.08) | 0.96 (0.71, 1.31) | 1.10 (0.94, 1.30) | 1.30 (1.01, 1.67)* |
|  |  |  |  |  |  |  |
| Diabetes^e^ | 161 (34.0) | 1.15 (0.96, 1.38) | 0.96 (0.75, 1.22) | 0.87 (0.56, 1.36) | 0.95 (0.76, 1.20) | 0.81 (0.54, 1.20) |
|  |  |  |  |  |  |  |
| Elevated hs-CRP (>3.0 mg/dL) | 212 (46.8) | 1.12 (0.97, 1.29) | 1.01 (0.84, 1.23) | 0.85 (0.60, 1.21) | 1.18 (0.98, 1.41) | 1.08 (0.79, 1.47) |

Abbreviations: *p,p*′-DDT, *p,p*′-dichlorodiphenyltrichloroethane; *p,p*′-DDE, *p,p*′-dichlorodiphenyldichloroethylene; HCB, hexachlorobenzene; β-HCH, β-hexachlorocyclohexane; HbA1c, glycated hemoglobin; HDL-C, high-density lipoprotein cholesterol; hs-CRP, high sensitivity C-reactive protein

Notes: Results expressed as Relative Risk (RR) and 95% confidence interval (CI) per 10-fold increase in OC pesticide exposure.

^a^ Adjusted for individual OC pesticides, age at outcome assessment (2022-2024), poverty status at exposure assessment (2009-2011), and years lived in the U.S. at exposure assessment (2009-2011)

^c^ Systolic blood pressure ≥130 mm Hg or diastolic blood pressure ≥ 85 mm Hg or drug treatment for high blood pressure

^d^ Metabolic Syndrome presence of ≥3 of: (1) waist circumference ≥88 cm; (2) serum triglycerides ≥150 mg/dL or drug treatment to lower cholesterol; (3) serum HDL-C<50 mg/dL or drug treatment to lower cholesterol; (4) systolic blood pressure ≥130 mm Hg or diastolic blood pressure ≥ 85 mm Hg or drug treatment for high blood pressure; (5) serum glucose ≥100 mg/dL or drug treatment for diabetes.

**^e^** Diabetes (self-report of drug treatment for diabetes, HbA1c ≥ 6.5%, glucose≥126 mg/dL)

* 95% Confidence Interval does not include the null hypothesis (0.0)

SUPPLEMENTARY FIGURE 1. DAG


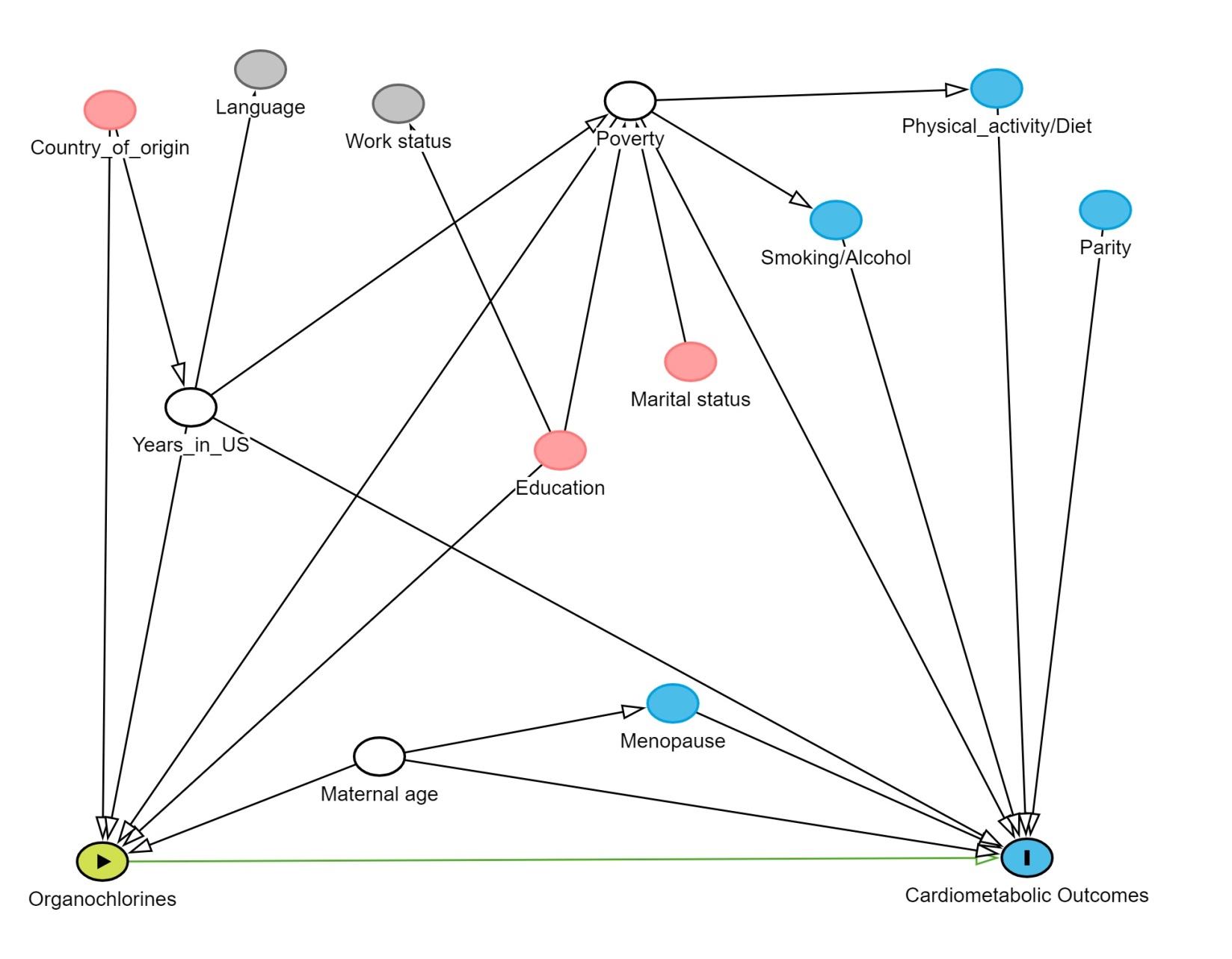

Supplement: Supplementary data [file NIHMS2120026-supplement-Supplementary_data.docx]
